# Cost-effectiveness of models for prevention of vertical HIV transmission – voluntary counseling and testing and choices of drug regimen

**DOI:** 10.1186/1478-7547-3-7

**Published:** 2005-07-18

**Authors:** Yot Teerawattananon, Theo Vos, Viroj Tangcharoensathien, Miranda Mugford

**Affiliations:** 1 International health Policy Program, Bureau of Policy and Strategy, Ministry of Public Health, Nonthaburi, Thailand; 2 Centre for Burden of Disease and Cost-Effectiveness, School of Population Health, University of Queensland, Brisbane, Australia; 3 School of Medicine, Health Policy and Practice, University of East Anglia, Norwich, UK

## Abstract

**Objectives:**

From a health care provider prospective, to assess the cost-effectiveness of four Antiretroviral therapy (ART) regimens given in addition to voluntary counselling and testing (VCT) for preventing mother-to-child transmission of HIV: a) Zidovudine (AZT); b) Nevirapine (NVP); c) a combination of AZT for early antenatal attenders and NVP for late arrivals; and d) combined administration of AZT and NVP and to assess the incremental cost-effectiveness of adding a second VCT session in late pregnancy.

**Design & Setting:**

We examine a hypothetical cohort of 100,000 pregnancies as a decision model. Cost and outcome parameters are estimated as they would apply under Thai routine health service conditions. Effectiveness probabilities are based on best available evidence, from systematic reviews where possible. The main outcome is the number of cases of paediatric HIV averted.

**Results:**

The combining administration of AZT and NVP is the most cost-effective drug option. One VCT session with AZT+NVP averts 337 cases of infection at 556 USD per case averted, while two VCT with the same drug regimen averts 16 additional cases at cost of 1,266 USD per infection averted. The incremental cost-effectiveness ratio of moving from 1VCT, AZT+NVP to 2VCT, AZT+NVP is 16,000 USD per additional averted case, which is much lower than the recommended threshold value for HIV infection averted in Thailand. Multivariate uncertainty analysis supports the findings, showing that at a threshold of 35,000 USD, 2VCT, AZT+NVP is preferable to other VCT and drug strategies.

**Conclusion:**

Interventions for preventing mother-to-child transmission of HIV are cost-effective. Further costs and negative effects of drug resistance, are unlikely to outweigh the social benefits of reduce transmission of HIV. This model suggests that the new drug regimen is a cost-effective option in the Thai health system at currently accepted thresholds for adopting health technologies.

## Introduction

Thailand is one of the countries having most success fighting the epidemic of HIV/AIDS infection [1,2]. Two randomised clinical trials conducted in Thailand have provided substantial impact on prevention of mother to child transmission of HIV/AIDS (PMTCT). The first demonstrated in 1999 that a short course of twice daily oral Zidovudine (AZT) was safe, well-tolerated and, in the absence of breast-feeding, lessened the risk for mother to child HIV-1 transmission from 18.9% to 9.4% [3]. This prompted the Thai Government to provide universal access to a short course of AZT in 2000 [4,5].

The second trial recently reported that a combination of AZT and a single dose of Nevirapine (NVP), administered both to the mother during labour and to the newborn, resulted in only two percent of children being born with HIV [6]. On release of these results, the Thai National Perinatal HIV Prevention programme incorporated this new regimen into its policy [7].

By 2004 "the current practice" is 300 mg oral AZT twice a day started at 28 week of gestation, a single dose of 200 mg NVP at onset of labour plus intra-labour 300 mg AZT orally every three hours until delivery. Newborns receive a single dose of NVP 6 mg after birth and AZT 2 mg per kg every six hours for 7 days if the mother received 4 or more weeks of AZT. The children born to mothers arriving late in pregnancy or to mothers who received AZT for less than 4 weeks, are given a six-week AZT regimen [7].

The Thai PMTCT programme provides free services for two rounds of Voluntary Counselling and Testing (VCT) for all pregnant women, at first antenatal (ANC) visit and at 28 weeks. The reason for the second VCT is to detect the newly HIV infected during pregnancy. HIV infected pregnant women receive free antiretroviral drugs, breast milk substitutes for 12 months and counselling with their partner to test their newborn at 12 and 18 months. The Ministry of Public Health (MOPH) purchases drugs and artificial milk in bulk and distributes it via its regional networks [8].

While modelling in a Sub-Saharan African setting [9], revealed that a single NVP dose provided to both mother and baby could be highly cost-effective in high sero-prevalence settings, the cost-effectiveness of NVP therapy in an Asian setting where prevalence is lower is unknown.

On the other hand, several studies have raised concern about the high rate of NVP resistance developing in mothers treated with a single regimen [10-13]. The rate was as high as 30–40% in South-Africa [14-16] and 17% in Thailand [17] and this would affect the choice of antiretroviral therapy (HAART) if the mother needs to be treated later on [18].

The purpose of this study is to appraise the cost-effectiveness of the regimen introduced in 2004, compared (regimen D in table 1) with several alternatives: 1) the previous Thai practice – a short course AZT regimen (regimen A); 2) the cheapest regimen consisting of a single NVP dose (regimen B); and 3) a mixed regimen of short course AZT for ANC arrivals at 34 weeks of gestation, and NVP for late arrivals beyond 34 weeks and for those who refuse the AZT regimen (regimen C). The last option is designed to maximise the effectiveness and minimise the problem of drug resistance. Furthermore, the analysis assesses the value of a second VCT round by comparing the cost-effectiveness of one and two maternal VCT for each of the four drugs options.

**Table 1:** Protocol of four drug regimens for health economic evaluation in Thai HIV transmission cost-effectiveness model

**Code**	**Drug regimens**	**Zidovudine (AZT)**		**Nevirapine (NVP)**	
		
		**mother**	**infant**	**mother**	**infant**
**A**	A short course AZT (practice in Thailand to 2004)	Starting from 32–34 week of gestation onward + intrapartum doses	From birth for 7 days (6 weeks in the case of the mother receiving <4 weeks AZT)	Not provided	Not provided
**B**	NVP alone (never adopted in the national policy)	Not provide	Not provide	Intrapartum single dose	Single dose after delivery
**C**	AZT or NVP (never adopted in the national policy)	Start at 32 but not latter than 34 weeks of gestations + intrapartum doses but not with NVP	From birth for 7 days (6 weeks in the case of the mother receiving <4 weeks AZT) – and given only the cases that mother received AZT	If mother know HIV status after 34 weeks then give single dose but not with AZT	Single dose after delivery if mother received NVP only
**D**	AZT and NVP (current practice, commencing from 2004)	Starting from 28 week of gestation onward + intrapartum doses	From birth for 7 days (6 weeks in the case of the mother receiving <4 weeks AZT)	In trapartum single dose	Single dose after delivery

## Design & Methods

### Study model

**Figure 1:**
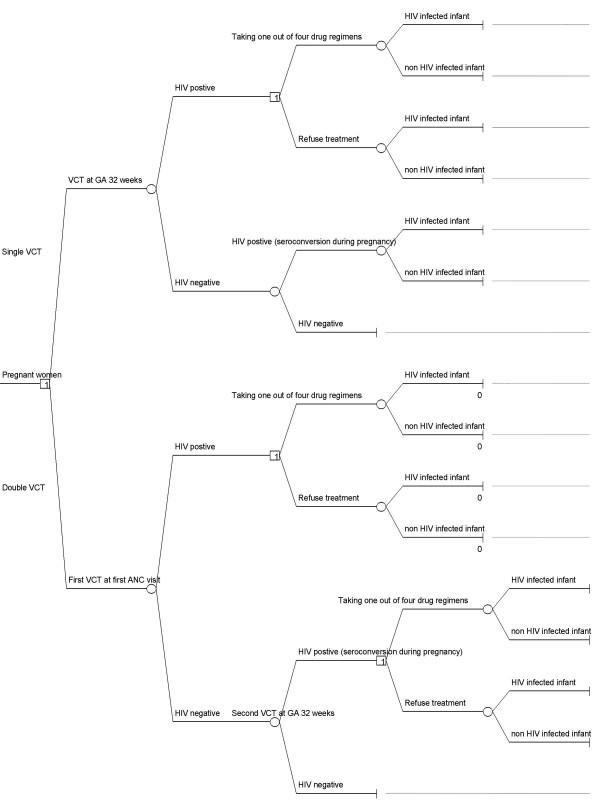
**The decision tree used to model the prevention of HIV vertical transmission. **(VCT = voluntary counseling and HIV testing, GA = gestational age).

We use a hypothetical cohort of 100,000 pregnancies as the study model. The decision tree in Figure 1 presents a flow of the programme options. Cost and outcome parameters are based on Thai settings. The combination of one and two VCT sessions and four antiretroviral therapy (ART) regimens leads to eight case options being considered within the model. For simplicity's sake we assign codes 1 and 2 for single and double VCT strategy, and A-D for four drug regimens.

Cost analysis is conducted from the perspective of the Ministry of Public Health (MOPH) as the Thai government pays all costs for VCT, ART and substitute feeding. We measure the programme outcomes as the net cost to the public-sector payer, total number of cases of paediatric HIV infection averted, and cost per paediatric HIV infection averted.

The cost-effectiveness of each intervention is calculated as (IC+AC-HC)/IA, where IC are the programme intervention costs, AC additional healthcare cost due to NVP resistance, HC the lifetime health care cost of an HIV infected infant (or cost offset), and IA the number of HIV infections averted by the intervention. We converted all cost and effectiveness at the present value (2003) with discounting of 5%.

### Input parameters (table 2)

**Table 2:** Estimated base-case values and their confidential intervals (CI) for input parameters in the Thai HIV cost-effectiveness model

**Parameters**	**Point estimate**	**95%CI for sensitivity analysis**	**Parameter distribution**	**Data sources**
**Epidemiology**				
Maternal HIV infection rate	1.5%			Ref.19
ANC after gestational age 34 weeks	7.4%	6.7–8.0%	Beta	Ref.8
Rate of perinatal HIV transmission	18.9%	13.2–24.4%	Beta	Ref.3
Rate of transmission via breastfeeding	12.0%	7.0–17.0%*	Beta	Ref.22
Percent of HIV infection detected by second VCT	4.7%	2.70–7.5%	Beta	Ref.20
Rate of HIV infected mothers who, treated with NVP, developed HIV resistance to NVP	17.4%	12.0–22.7%	Beta	Ref.17
Rate of HIV infected mothers who need to be treated AIDS within a year after delivery	33.9%	29.6–38.3%	Beta	Ref.28
**Efficacy of Antiretrovial therapy**				
Odds of transmitting the virus when mother received AZT > = 4 weeks versus placebo	0.46	0.35–0.60	Normal	Ref.23
Odds of transmitting the virus when mother received AZT < 4 weeks versus receiving AZT > = 4 weeks	1.40	0.82–2.38	Normal	Ref.24
Risk of transmitting the virus with NVP regimen versus placebo	0.51	0.33–0.79	Normal	Ref. 23
Risk of transmitting the virus with AZT+NVP regimen versus receiving AZT > = 4 weeks	0.23	0.05–0.41	Normal	Ref.6
**Compliance to the Programme**				
Infected pregnant women who know their HIV status before or at 36 week of gestation and accept AZT	75%	70–90%	Beta	Ref.8
Infected pregnant women who know their HIV status after 36 week of gestation and accept AZT	65%	55–90%*	Beta	Assumption (see text)
Infected pregnant women who know their HIV status before or at 36 week of gestation, do not accept AZT but accept NVP	50%	30–70%*	Beta	Assumption (see text)
Infected pregnant women who know their HIV status before or at 36 week of gestation and accept NVP	85%	70–90%*	Beta	Assumption (see text)
Infected pregnant women who know their HIV status after 36 week of gestation and accept NVP	75%	70–90%*	Beta	Assumption (see text)
Infected pregnant women who know their HIV status before or at 36 week of gestation and accept AZT+NVP	84%	80–90%	Beta	Ref.17
Infected pregnant women who know their HIV status after 36 week of gestation and accept AZT+NVP	75%	70–80%*	Beta	Assumption (see text)
**Programme unit cost**	**US$ 2003**			
VCT for HIV negative pregnancy	2.69	1.57–7.79	Gramma	Ref. 8
VCT for HIV positive pregnancy	7.10	3.82–14.54	Gramma	Ref. 8
HIV testing for baby born by infected mother	5.61	3.18–11.65	Gramma	Ref. 8
Cost of antepartum AZT (per weeks)	10.50			Thai Department of Health
Cost of intrapartum AZT	2.30			Thai Department of Health
Cost of infant AZT (per week)	17.20			Thai Department of Health
Cost of NPV for mother and infant	3.10			Price survey by authors
Breast milk substitutes (per 1 year)	175.90			Thai Department of Health
Incremental cost of switching from NNRTI-base treatment regimen to PI-based regimen	497	147–847	Gramma	Ref.29
**Public sector health expenditure**				
Life time pediatric HIV/AIDS treatment cost	1,680	1,340–2,015	Gramma	Ref.30

Note that a range for sensitivity analysis derived from 95% CI of each parameter distribution except * that based on assumption

For each parameter we determine base-case values. A maternal HIV infection rate of 1.5% was reported by national sentinel surveillance 2001 [19]. A prospective mother-to-child study from 1992 to 1994 in Bangkok [20] found that with a second VCT round during pregnancy an additional 4.7% (95% CI 2.7% and 7.5%) HIV positive mothers were detected who had become infected during pregnancy. In other word, we assume the second VCT picks up 4.7% of the incidence cases.

In Thailand, all pregnant women have at least one ANC visit during pregnancy [21]. As it takes two weeks to know the maternal HIV status the 7.4% of pregnant women arriving late for ANC are likely to receive ART later than 36 weeks compromising the optimal period for effective AZT treatment of four weeks [8].

The risk of perinatal transmission without treatment is 18.9% [3] and with breast-feeding is assumed to be an additional 12% [22]. Lallemant et al [6] report that the odds ratio of transmitting the virus by regimen D versus control (no prevention) is 0.23 (95% CI 0.05 to 0.41). The effectiveness of other drug options is derived from a Cochrane systematic review [23] indicating an odds ratio compared to placebo of 0.46 (95% CI 0.35 to 0.60) for regimen A and 0.51 (95%CI 0.33 to 0.79) for regimen B.

To estimate the risk of transmission among late arrivals for ANC who get AZT treatment less than 4 weeks in regimen A, we apply an odds ratio of 1.40 (95% CI 0.82 to 2.38) found in a Thai study [24] comparing the risk of transmission between a short (from 36 weeks onwards) and long (from 28 weeks) maternal course of AZT. In the absence of evidence of the efficacy of regimen D started after 28 weeks but before 36 weeks of gestation, we assume the same efficacy if treatment is started before 34 weeks, and the lower efficacy of regimen A for those starting after 34 weeks.

In 13 provinces in the North of Thailand [8] 75% of infected pregnant women accepted AZT treatment after knowing their HIV status. A recent study found a higher proportion (84%) of pregnant women accepted regimen D [17]. We use the former figure as the base case scenario for infected women who knew their HIV status before 36 week of gestation and accepted AZT treatment in regimens A and C, and the latter for regimen D.

As we know that women make their first antenatal visit late in pregnancy tend to report low education and socioeconomic status [25]. We, therefore, assume a lower proportion of 65% of infected pregnant women who know their HIV status after 36 weeks accept AZT in programmes A and C, and 75% of those accept AZT and NVP in programme D. For those who refuse AZT treatment for whatever reason, we assume 50% would accept the simpler regimen of NVP in programme C.

For the regimen of single NVP, we propose 85% of infected pregnant women who know their serologic status before 36 weeks and 75% thereafter enroll in the programme B. This enrollment rate is slightly higher than with AZT as drug administration is simpler.

The study presents all prices in US$ (USD) at 2003 price units. Costs of providing VCT, maternal and infant antiretroviral treatment are derived from 160 MOPH hospitals [8]. Briefly, data on the units of each category of resources used and valued were gathered by mean of a questionnaire sent to each hospital participating in the study. Intervention unit costs include recurrent labour and non-labour expenditures but exclude capital depreciation.

A study in Thailand found that 17.4% (95% CI 12.0% to 22.7%) of infected mothers who were treated with a single dose NVP developed a strain of HIV resistant to the drug [17]. Several studies report that the resistant virus spontaneously reverts to a wide-type genotype by one year [14,26], meaning that the resistance would not have altered effectiveness of prevention in further pregnancies. Nevertheless, the concern is that those who were resistant and were then treated for AIDS following delivery would have a much more difficult time controlling the virus [13,18]. AIDS experts therefore recommend a more expensive treatment regimen based on protease inhibitors (PI) as a first line treatment for mothers with the NVP resistant virus instead of the common and cheaper regimen, based on Non-Nucleoside Reverse Transcriptase Inhibitors (NNRTI) [27]. A report from MOPH [28] reveals 33.9% of infected mothers need treatment for AIDS by a year after delivery and the incremental cost of switching from the cheaper to the more expensive treatment regimen is 497 USD (95% 147 USD to 847 USD) assuming the incremental cost occurs only for the first three years of the treatment [29].

Assuming 80% of lifetime paediatric HIV/AIDS treatment cost is shouldered by the public sector, the lifetime medical care cost was estimated at 3,300 USD in 1997 (25 Baht per 1 USD) [30]. Converting the 1997 figures to 2003 values using the general consumer price index, the public sector health expenditure for a case of paediatric AIDS is 1,680 USD (40 Baht per 1 USD). A range of 1,340–2,015 USD is proposed for sensitivity analysis. A clinical trial of morbidity and mortality among breast fed and formula fed infants of HIV-1-infected women in Kenya found a similar overall mortality rate, incidence of diarrhea, pneumonia and other serious complications among the two groups [31]. Therefore, we do not add costs of treatments for excess diarrhoea and respiratory tract infections among formula-fed infants.

### Uncertainty analysis

To handle uncertainty in the model input parameters of interest are ascribed a distribution that reflects the uncertainty associated with their true value (table 2) and entered in a probabilistic uncertainty analysis. For example, the beta-distribution was the choice of distribution for probability parameters which were bounded zero-one and the gamma distribution was modelled for unit cost parameters [32].

The results from 1,000 calculations are presented in a CE acceptability curve based on the concept of net-benefit approach suggested by Stinnett and Mullahy [33] and Briggs et al [34]. It shows the probability in the 1,000 iterations of the model that any of the eight interventions is the most cost-effective option given different willingness to pay thresholds.

## Results

**Figure 2:**
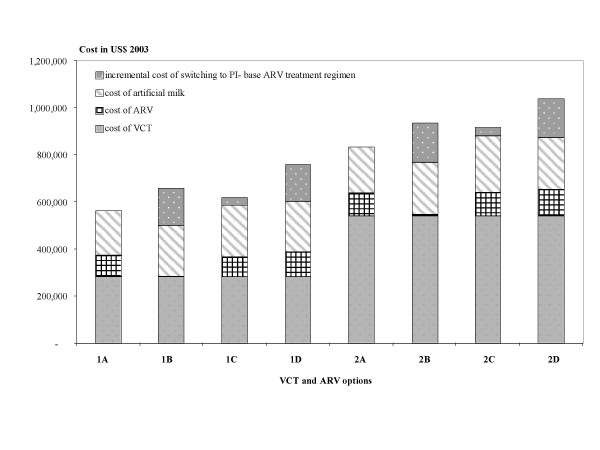
Total programme cost component by voluntary counselling and HIV testing (VCT) and antiretroviral drug (ARV) options.

The total programme cost, including costs of VCT, ART, artificial milk and dealing with drug resistance, of option 1A is the cheapest; option 2D is the most expensive, mainly due to the costs of second VCT and dealing with the problem of drug resistance (Table 3 and Figure 2).

**Table 3:** Costs, effectiveness and cost-effectiveness of 6 intervention options for the Thai HIV cost-effectiveness model, US$ 2003

**Programme model**	**1A**	**1B**	**1C**	**1D**	**2A**	**2B**	**2C**	**2D**
Programme cost	560,000	500,000	580,000	600,000	840,000	770,000	880,000	880,000
Incremental cost of switching NNRTI-base treatment to PI-base treatment		160,000	30,000	150,000		160,000	30,000	160,000
Total programme cost	560,000	650,000	610,000	750,000	840,000	930,000	920,000	1,040,000
Life time treatment cost for pediatric HIV/AIDS	390,000	430,000	460,000	560,000	410,000	450,000	500,000	590,000
**Net programme cost**	170,000	220,000	160,000	190,000	430,000	480,000	410,000	450,000
Number of infections averted by the program	233	258	273	337	245	271	300	353
**Cost-effectiveness ratio per averted infection**	716	851	570	556	1,740	1,776	1,381	1,266

Note: numbers for programme costs are given to nearest 10,000 US$, 2003 price levels

Options 1C and 2C have the lowest net programme cost in comparison to the other drug regimens, while 1B and 2B are the most expensive alternatives, on account of the higher cost of dealing with drug resistance and smaller offset-cost.

The mixed regimen of AZT and NVP with 2 VCT sessions (programme 2D) is the most effective strategy, averting 353 infections. Compared to programme 1D, the second VCT session prevents a small number of 16 (353–337) additional cases at the additional cost of 260,000 USDThe least effective regimens are those based on AZT only, followed by the single NVP regimens (1B and 2B) which prevent 25 and 26 extra infections compared to the AZT only regimens (1A and 2A).

### Cost-effectiveness

Compared with no routine prophylactic ART, programme 1D is the most cost-effective regimen costing 556 USD to avert one paediatric HIV infection. Option 2B is the least cost-effective regimen at more than three times the cost per additional case prevented.

With relative little benefit gained from the second VCT, all regimens with the single VCT strategy are more cost-effective than those with 2 VCT sessions.

### Uncertainty analysis

**Figure 3:**
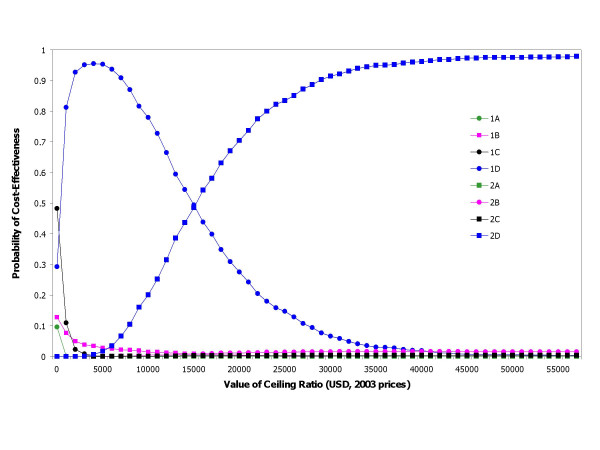
**Acceptability curves using net-monetary benefit approach for the choice of prevention strategy. **The proportion of simulations in which a strategy has the highest net-benefit across all strategies among 1,000 replications of the model (sum of all probability at each maximum willingness to pay for one HIV infection averted or "ceiling ratio" equal 1).

We plotted acceptability curves using net-monetary benefit approach for the choice of prevention strategy in figure 3. To explain this, we consider table 3 where the most effective drug option is programme D. The incremental cost of moving from 1D to 2D is 260,000 (450,000-190,000) USD for 16 (353-337) additional infections averted. In other words, the incremental cost-effectiveness ratio of giving up the 1D regimen and adopting 2D is 16,000 USD per additional infection averted. In Figure 3 this incremental cost-effectiveness value is represented by showing the line of 1D crossed the line of 2D at the ceiling ratio of 15,000USD (they were not exactly the same value since the first is a deterministic and the latter is a probabilistic value). If the decision maker would prefer a confidence level greater than 95%, the threshold is 35,000 USD per infection averted. Further studies would be needed to improve the accuracy of the cost-effectiveness results. In particular, better information on the proportion of infected pregnancy detected by second VCT and the cost of VCT would improve the model because these parameters have the greatest bearing on uncertainty.

**Figure 4:**
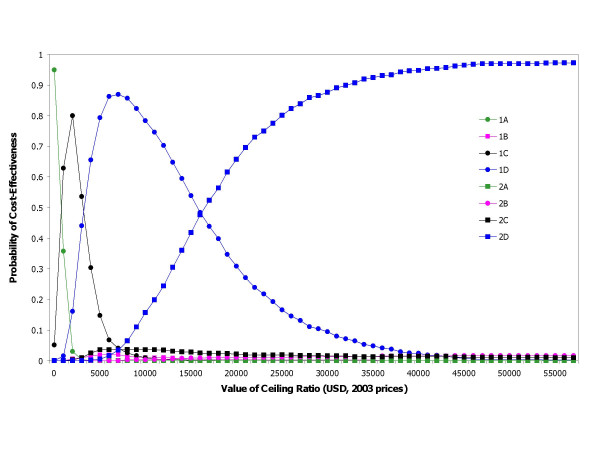
Acceptability curves for the choice of prevention strategy at 40% of rate of HIV resistant to Nevirapine.

Having mentioned above that the rate of developing NVP resistant virus among Thais was considerably lower than of Sub-Saharan setting, we therefore tested the results assuming a rate of developing HIV resistant to NVP as high as in a Sub-Saharan setting (40%). Figure 4 illustrates that the regimens that contains no NVP (A) or contains less NVP (C) are dominant. Programme 2D is still to be the preferable choice if the willingness to pay threshold greater than 40,000 USD per HIV infection averted with the statistical confidence level 95%. It is interesting to note that 1A is a significantly dominant regimen at the lowest threshold of zero willingness to pay, that is, where a decision has been made that no further resources would be attributed to healthcare.

## Discussion

This study has presented an economic evaluation of a comprehensive range of VCT and choices of drug regimen for PMTCT in Thailand. We excluded a long course of AZT, ACTG 076, in our assessment since it is relatively complicated (given intravenous form of AZT to mother during labour) and expensive, and may be difficult to adopt in developing countries [35,36].

There are two major policy concerns in this study: whether to recommend single or double VCT, and which of four drug regimens is the most cost-effective investment per infection averted in a setting with moderately high HIV sero-prevalence in pregnancy. The assessment has proved by both point estimate and multivariate uncertainty analysis that the programme D has a lower cost per paediatric HIV infection averted than other alternatives.

For the choice of VCT, though, we found that 1VCT is less costly than 2VCT. International experience with the accepted cost-utility ratios suggests that cost per Life Year Gained (LYG) or Quality Adjusted Life Year (QALY) threshold is 3 × per capita GDP [37]. This application would presently lead to a threshold value in Thailand of between 21,000 USD per LYG or QALY or 407,000 USD per paediatric HIV infection averted (assuming 19.4 LYG per HIV infection averted and the current practice, programme 2D, clearly represents value for money. Thus, our evidence supports the new policy of the Thai National Perinatal HIV Prevention programme having introduced programme 2D as a national regimen for PMTCT.

This study is partly compatible with one conducted in Mexico, another low HIV prevalence setting, [38]. Both studies similarly identify that VCT has a major share of total programme cost but also is essential to the efficacy of the ART programme [39]. Minimisation of PMTCT cost in low-prevalence setting should therefore focus on VCT costs rather than drug cost. However, the result of this study provides additional information that providing artificial milk and dealing with the problem of drug resistance also add a considerable cost.

Because the authors chose to explore costs and outcomes of the PMTCT in particular context of Thai setting and use only government perspective, applying these findings to somewhere else or using other viewpoints should be done with cautious. For example, the rate of accepting VCT in Thailand may be much higher than in other countries. Also, the percentage of detecting HIV infection by the second VCT used in this study is rather high. However, this study offers a useful and comprehensive framework for evaluation of the PMTCT, especially in developing countries where resources do not permit adequate development of basic health need but shoulder a major burden of HIV/AIDS.
